# Region- and cell type-specific changes in gene expression in the cerebellum after classical fear conditioning

**DOI:** 10.21203/rs.3.rs-6469280/v1

**Published:** 2025-05-07

**Authors:** Yong-Seok Lee, Jungeun Ji, Jinhee Baek, Kyoung-Doo Hwang, Seunghwan Choi, Ted Abel, Joon-Yong An, Junko Kasuya

**Affiliations:** Seoul National University College of Medicine; University of Iowa; University of Iowa; University of Iowa; University of Iowa; University of Iowa; Korea University; University of Iowa

**Keywords:** spatial transcriptomics, single-nucleus RNA-seq, cerebellum, deep cerebellar nuclei, Purkinje cell layer, fear memory

## Abstract

The cerebellum has recently been recognized for its role in non-motor functions, including classical fear conditioning. However, the molecular mechanisms underlying non-motor learning and memory remain largely unknown. Here, we investigate the transcriptional changes in the cerebellum associated with auditory fear conditioning. Spatial transcriptomic analysis revealed that in the deep cerebellar nuclei (DCN), an output region of the cerebellum, the expression of immediate early genes increased following fear learning and retrieval, suggesting that DCN may contribute to fear memory processing. As for the cerebellar cortex, robust and region-specific transcriptional changes were observed, with distinct expression patterns emerging across the Purkinje cell layer of vermis region. To further elucidate transcriptional changes in specific DCN cell types involved in fear processing, we performed single-nucleus RNA sequencing and identified prominent gene expression changes in *Kit* + inhibitory neurons. Collectively, our findings highlight region- and cell-type-specific molecular adaptations in the cerebellum, providing insights into its contribution to non-motor learning.

## Introduction

The cerebellum is a key brain region involved in modulating a wide range of behavioral outputs, including not only motor but also non-motor functions, such as fear learning and social behaviors^1–8^. Although traditionally thought to process sensorimotor information through cerebellar neuron types (e.g., Purkinje cells and granule cells) that are seemingly uniform across the cerebellum, recent studies reveal substantial molecular, structural, and physiological heterogeneity within the cerebellum^9–17^. This heterogeneity suggests that spatially distinct cerebellar cell populations exhibit different activity patterns in response to learning, depending on their anatomical location and molecular characteristics.

Classical fear conditioning is a well-established form of non-motor associative learning that involves the cerebellum^4,6,7,18–20^. Previous studies have demonstrated that different cerebellar circuits and subregions regulate various aspects and temporal stages of fear learning^6,18,20–25^ and that fear conditioning induces synaptic plasticity, requiring molecular modifications at multiple cerebellar synapses^18^. However, how classical fear conditioning drives molecular changes in the cerebellum remains unclear.

In contrast, it is well known that cerebellum-dependent motor learnings induce transcriptional and translational changes in cerebellar circuits^13,26,27^. For instance, oculomotor learning tasks—such as the vestibulo-ocular reflex and optokinetic response —promote learning-dependent protein synthesis in the flocculus^26^, while delayed eyeblink conditioning alters transcriptional profiles in the anterior interpositus nucleus across different learning stages^27^. Moreover, a subset of cerebellar Purkinje cells expressing *Plcb4* + undergoes transcriptional plasticity during motor learning and is essential for both associative and motor learning through fibroblast growth factor signaling^13^. Despite accumulating evidence suggesting that the cerebellum also plays a role in non-motor learning and memory, such as classical fear conditioning, how non-motor stimuli reshape the cerebellar molecular architecture remains unknown.

To address this question, we employed spatial transcriptomics and single-nucleus RNA sequencing (snRNA-seq) to investigate how classical auditory fear conditioning modulates cerebellar gene expression across different learning phases in male mice. Our analysis revealed distinct transcriptional responses in the cerebellar vermis, robust induction of immediate early genes in the DCN (deep cerebellar nuclei) after fear memory acquisition and retrieval. Furthermore, a specific subset of *Kit* + inhibitory DCN neurons displayed pronounced transcriptional alterations, including upregulation of mGluR5, suggesting the formation of an engram through inhibitory signaling. These findings uncover a heterogeneous molecular landscape underlying non-motor associative learning in the cerebellum.

## Results

### Spatial transcriptomic analysis in the cerebellum after classical fear conditioning

**Figure 1:**
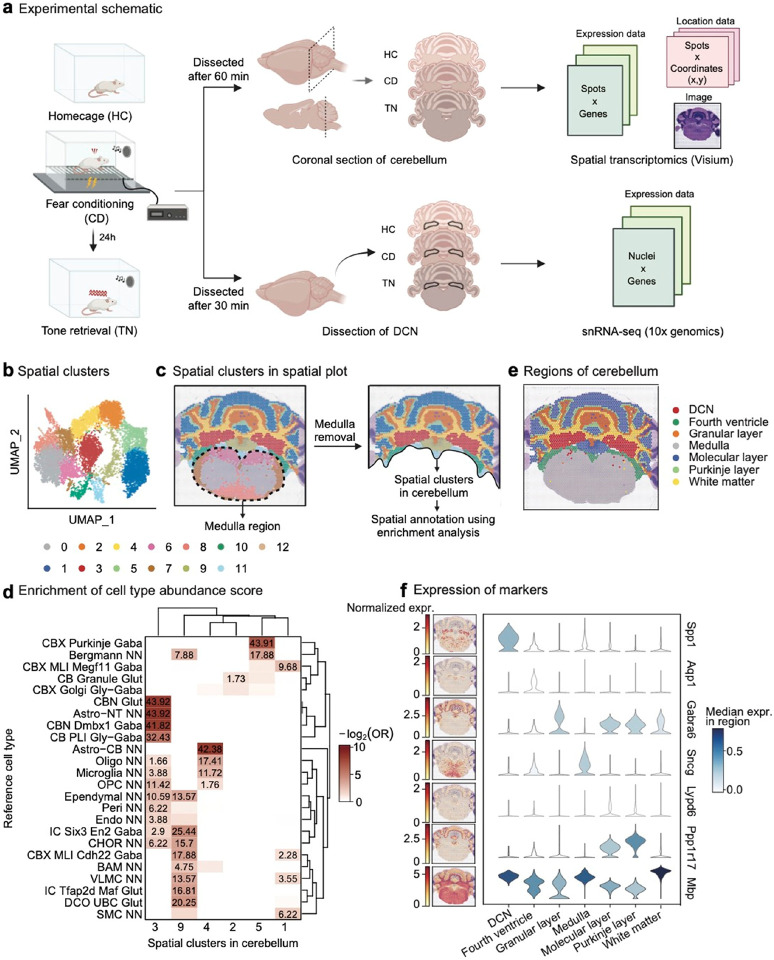
Spatial transcriptomic profile of the mouse cerebellum after fear conditioning **a**, Experimental overview. During classical fear conditioning, mice underwent five tone-footshock pairings (2.8 kHz, 80 dB, 30 s tone; 0.5 mA, 2 s footshock). Brain samples were obtained 60 min after behavior tests for spatial transcriptomics, and 30min after for single nucleus sequencing. A coronal section from a single mouse per condition was used for spatial transcriptomic analysis, while DCNs from five mice per condition were pooled for snRNA-seq. **b**, UMAP representation of the dataset, colored by spatial clusters (9,780 spots, n=1 mouse per condition). **c**, Spatial plot of the dataset, colored by spatial clusters, illustrating the schematic process of medulla removal to retain only the cerebellar regions. **d**, Heatmap of enrichment analysis comparing spatial clusters to reference snRNA-seq cerebellar cell types. Cell type abundance scores for each spot, estimated using cell2location, were binarized and analyzed using Fisher’s exact test. Significance is represented as −log_10_(FDR) values for results meeting the FDR < 0.05 threshold, with Log_2_(OR) indicated by color. Abbreviations: CBX, cerebellar cortex; MLI, molecular layer interneurons; CB, cerebellum; PLI, Purkinje cell layer interneuron; OPC, oligodendrocyte precursor cells; Peri, pericytes; Endo, endothelial cells; IC, inferior colliculus; CHOR, choroid plexus; BAM, border-associated macrophages; VLMC, vascular leptomeningeal cells; DCN, deep cerebellar nuclei; UBC, unipolar brush cells; SMC, smooth muscle cells; NN, non-neuronal. **e**, Spatial plot of the dataset, colored by annotated regions. **f**, Expression of marker genes of each cerebellar region, visualized using a spatial plot (left) and violin plot (right).

To investigate region-specific gene expression changes in the cerebellum across different phases of auditory fear conditioning, we performed spatial transcriptomics (10X Visium) on coronal sections of the mouse cerebellum, collected 1 h after fear conditioning (conditioned, CD) or 1 h after auditory memory retrieval (tone retrieval, TN). Mice that remained in their home cage (HC) were sacrificed at a similar circadian time and served as controls (Fig. 1a). Each sample contained between 3,000 and 3,500 spatial spots, yielding an integrated dataset of 9,780 spots and 18,779 genes. Unsupervised clustering, based on both gene expression profiles and spatial coordinate values identified 13 distinct clusters (Fig. 1b, c). Due to the complexity of the dissection process, our samples included portions of the medulla. To refine our analysis and focus specifically on the cerebellum, we selected only the clusters corresponding to cerebellar regions (Fig. 1c) for downstream analysis.

We then performed cell type enrichment analysis using a well-annotated reference scRNA-seq dataset^28^ (Fig. 1d, **Extended Data** Fig. 1a, **Supplementary Table 1a**). Additionally, we manually compared our data with an *in situ* hybridization reference dataset^29^ to identify non-cerebellar regions and those not clearly defined using the enrichment analysis (**Extended Data** Fig. 1b). Our 13 spatial clusters were classified into seven distinct regions: three cerebellar cortical regions (molecular layer, granular layer and Purkinje cell layer), DCN, white matter, fourth ventricle, and medulla (Fig. 1e). The expression patterns of well-known cell type markers confirmed the accuracy of our regional annotations (Fig. 1f). Furthermore, all three experimental conditions exhibited similar distributions across these regions (**Extended Data** Fig. 1c, d), suggesting that fear conditioning does not alter the overall regional composition of cerebellar cell populations.

### Induction of immediate early genes in DCN

Immediate early genes (IEGs), such as *cFos* and *Egr1*, are well-established markers of neuronal activity^30,31^. Although prior studies have explored behavior-induced IEGs in the cerebellum^32^, which IEGs consistently serve as reliable markers of activity within this brain region remains unclear.

**Figure 2:**
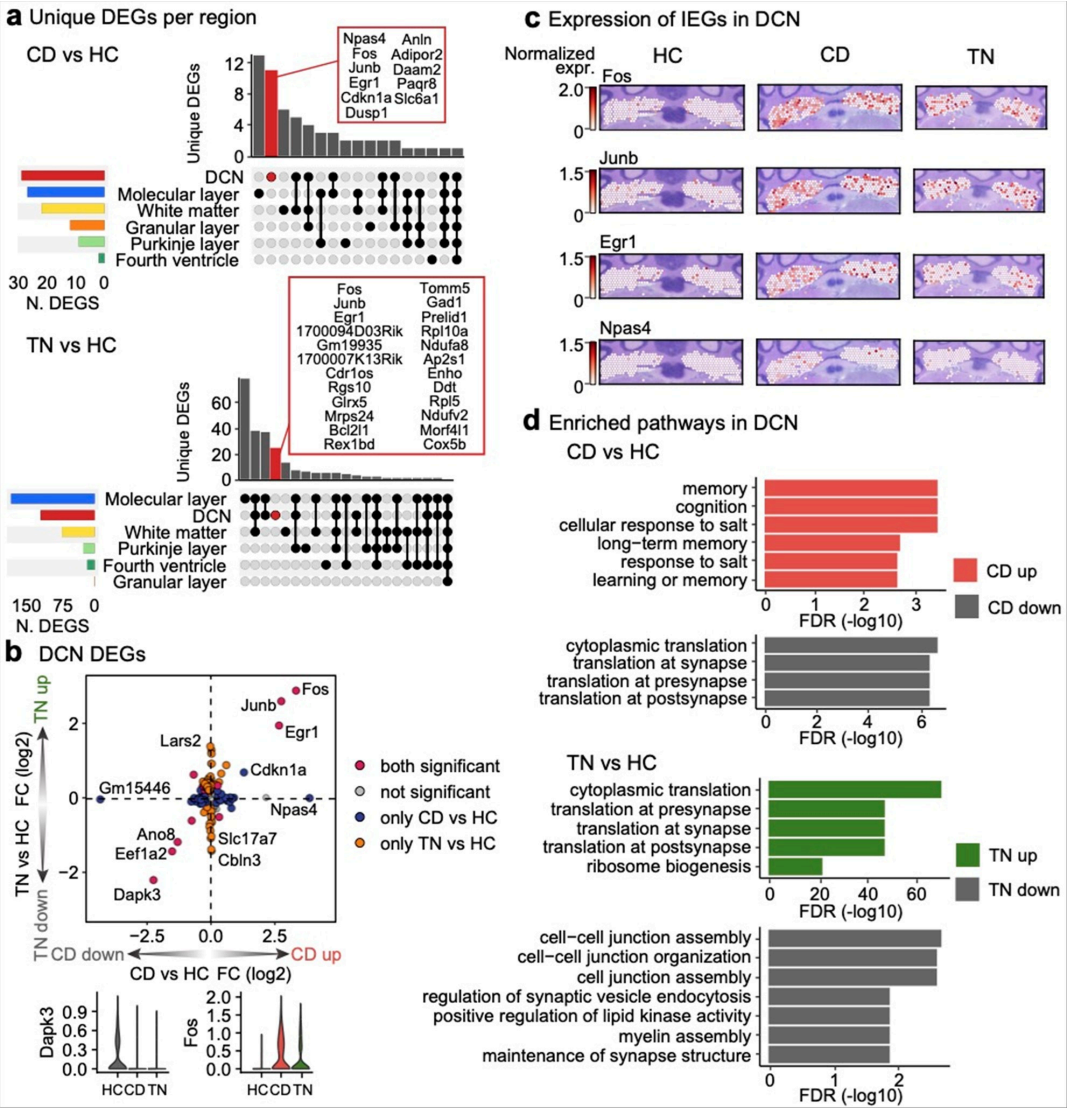
Different gene expression signatures in the DCN during fear learning **a**, UpSet plots showing unique and shard DEGs upregulated during the CD vs. HC comparison (top) and the TN vs. HC comparison (bottom) across cerebellar regions. The column highlighting DCN-specific DEGs is marked in red. **b**, Quadrant plot illustrating log_2_FC values for DCN DEGs in the CD vs. HC (x-axis) and TN vs. HC comparisons (y-axis). Genes are colored based on the comparison in which they are significant. Violin plots show normalized expression patterns of the top “both significant” DEGs in quadrant I (left) and quadrant III (right), separated by condition. **c**, Spatial plots showing normalized expression of IEGs *Fos*, *Junb*, *Egr1*, and *Npas4* in the DCN. **d**, Gene Ontology (GO) enrichment analysis results for the CD vs. HC (top) and TN vs. HC comparisons (bottom) in oligodendrocytes. Bar colors indicate the direction of enrichment, and false discovery rate (FDR) values denote significance.

To investigate the transcriptional signature of fear learning in each cerebellar region, we conducted a region-specific differentially expressed genes (DEG) analysis in the cerebellum across conditions HC, CD, and TN. Likelihood ratio test (LRT) revealed that the molecular layer exhibited the most significant gene expression changes across all conditions, followed by the DCN (**Extended Data** Fig. 2, **Supplementary Table 2a**). We next performed pairwise comparisons between the three conditions (CD vs. HC, TN vs. HC, and TN vs. CD) to identify genes regulated during specific phases of fear learning. Notably, the DCN was the only region where IEGs *Fos*, *Junb*, *Egr1*, and *Npas4* were significantly upregulated during fear learning (Fig. 2a). Among these, *Fos*, *Junb*, and *Egr1* were the most significantly upregulated DEGs during both the CD and TN phases (Fig. 2b). This indicates enhanced neural activation in the DCN during both fear memory acquisition and consolidation. Interestingly, *Npas4* was identified as the top upregulated DEG in the CD vs. HC comparison but did not show significant changes in the TN vs. HC comparison (Fig. 2b). This suggests that *Npas4* may play a selective role in fear memory acquisition but not in memory retrieval within the DCN. The enriched expression patterns of IEGs during fear learning were further confirmed through spatial visualization (Fig. 2c).

Functional analysis of the DEGs whose expression was higher in the DCN during the CD phase than during the HC phase revealed enrichment for fear memory-related processes, including memory, cognition, long-term memory, and regulation of synaptic plasticity (Fig. 2d, **Supplementary Table 2b**). CD-upregulated DEGs involved in memory and cognition included *Calb1*, *Egr1*, *Npas4*, *S100b*, and *Slc61a*. In contrast, the DEGs whose expression was higher during the TN phase than during the HC phase were enriched in processes related to cytoplasmic translation at the synapse (Fig. 2d, **Supplementary Table 2b**).

Overall, our findings demonstrate region-specific neuronal activation within the DCN following fear conditioning and reveal distinctive transcriptional patterns throughout the progressive stages of fear memory formation and retrieval.

### Validation of IEG expression in the DCN

**Figure 3:**
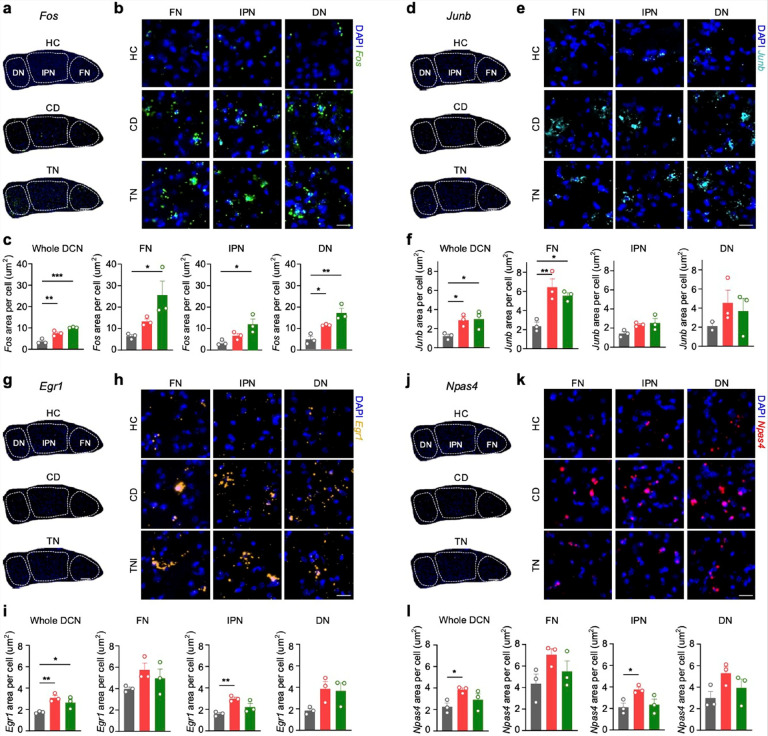
Immediate early genes are upregulated in the DCN during fear conditioning **a**, ISH results showing the expression of *Fos* in the DCN subnuclei. (FN, fastigial nucleus; IPN, interpositus nucleus; and DN, dentate nucleus). Scale bar: 150 μm. **b**,Representative ISH images of *Fos* expression in each subnucleus. Scale bar: 20 μm. **c**, Quantification of ISH data for *Fos* (n = 3 per group). Whole DCN: One-way ANOVA: F(2,6) = 33.12, *P* = 0.0006. Dunnett’s multiple comparisons test: **P = 0.0059, ***P = 0.0004. FN: One-way ANOVA: F(2,6) = 6.258, *P* = 0.0340. Dunnett’s multiple comparisons test: *P* = 0.4005 (HC vs. CD), *P = 0.0229 (HC vs. TN). IPN: One-way ANOVA: F(2,6) = 7.529, *P* = 0.0231. Dunnett’s multiple comparisons test: *P* = 0.3211 (HC vs. CD), *P = 0.0152 (HC vs. CD). DN: One-way ANOVA: F(2,6) = 17.83, *P* = 0.0030. Dunnett’s multiple comparisons test: *P = 0.0327 (HC vs. CD), **P = 0.0018 (HC vs. TN). **d**, ISH results showing *Junb* expression in the DCN. Scale bar: 150 μm. **e**, Representative ISH images for *Junb* in each subnucleus. Scale bar: 20 μm. **f**, Quantification of ISH data for *Junb*: Whole DCN: One-way ANOVA: F(2,6) = 6.243, *P* = 0.0342. Dunnett’s multiple comparisons test: *P = 0.0452 (HC vs. CD), *P = 0.0341 (HC vs. TN). FN: One-way ANOVA: F(2,6) = 13.50, *P* = 0.0060. Dunnett’s multiple comparisons test: **P = 0.0046 (HC vs. CD), *P = 0.0151 (HC vs. TN). IPN: One-way ANOVA: F(2,6) = 3.999, *P* = 0.0788. Dunnett’s multiple comparisons test: *P* = 0.1167 (HC vs. CD), *P* = 0.0677 (HC vs. TN). DN: One-way ANOVA: F(2,5) = 0.8278, *P* = 0.4892. Dunnett’s multiple comparisons test: *P* = 0.3871 (HC vs. CD), *P* = 0.6351 (HC vs. TN). g, ISH results showing *Egr1* expression in the DCN. Scale bar: 150 μm. **h**, Representative RNAscope images of *Egr1* expression in each subnucleus. Scale bar: 20 μm. **I**, Quantification of ISH data for *Egr1*expression: Whole DCN: One-way ANOVA: F(2,6) = 9.602, *P* = 0.0135. Dunnett’s multiple comparisons test: **P = 0.0091 (HC vs. CD), *P = 0.0476 (HC vs. TN). FN: One-way ANOVA: F(2,6) = 2.043, *P* = 0.2105. Dunnett’s multiple comparisons test: *P* = 0.1520 (HC vs. CD), *P* = 0.4686 (HC vs. TN). IPN: One-way ANOVA: F(2,6) = 10.76, *P* = 0.0104. Dunnett’s multiple comparisons test: **P = 0.0064 (HC vs. CD), *P* = 0.1345 (HC vs. TN). DN: One-way ANOVA: F(2,6) = 3.630, *P* = 0.0927. Dunnett’s multiple comparisons test: *P* = 0.0863 (HC vs. CD), *P* = 0.1185 (HC vs. TN). **j**, ISH results showing *Npas4* expression in the DCN. Scale bar: 150 μm. **k**, Representative ISH images of *Npas4*expression in each subnucleus. Scale bar: 20 μm. **l** Quantification of ISH data for *Npas4*: Whole DCN: One-way ANOVA: F(2,6) = 4.204, *P* = 0.0722. Dunnett’s multiple comparisons test: *P = 0.0486 (HC vs. CD), *P* = 0.4340 (HC vs. TN). FN: One-way ANOVA: F(2,6) = 2.685, *P* = 0.1469. Dunnett’s multiple comparisons test: *P* = 0.1030 (HC vs. CD), *P* = 0.5437 (HC vs. TN). IPN: One-way ANOVA: F(2,6) = 5.519, *P* = 0.0437. Dunnett’s multiple comparisons test: *P = 0.0380 (HC vs. CD), *P* = 0.8627 (HC vs. TN). DN: One-way ANOVA: F(2,6) = 2.836, *P* = 0.1358. Dunnett’s multiple comparisons test: *P* = 0.0955 (HC vs. CD), *P* = 0.5664 (HC vs. TN).

To validate the IEG data obtained from spatial transcriptomics, we performed fluorescence *in situ* hybridization (ISH) after fear conditioning and retrieval testing. The expression of IEGs *Fos*, *Junb*, *Egr1*, and *Npas4*, known markers of neuronal plasticity, was significantly increased in the DCN following fear learning or retrieval tests (Fig. 3). Interestingly, the expression of *Fos*, *Junb*, and *Egr1* was elevated after both fear conditioning and retrieval (Fig. 3a–i), whereas that of *Npas4* was elevated only after fear conditioning (Fig. 3j–l). These results align with the patterns observed in the spatial transcriptomics data. The DCN is composed of three subnuclei: the fastigial nucleus (FN), interpositus nucleus (IPN), and dentate nucleus (DN). We compared the IEG expression levels among these subnuclei and found distinct expression patterns. For instance, *Fos* was upregulated across all three subnuclei (Fig. 3a–c), whereas *Junb* showed a significant increase only in the FN (Fig. 3d–f). These findings suggest that IEGs may play distinct roles in fear learning through their respective downstream targets. Interestingly, such increased expression of IEGs was not observed in the cerebellar cortex (**Extended Data Fig. 7**).

Our validation experiments confirm that IEGs are prominently induced in the DCN during fear conditioning^4,6,21,33^, with distinct expression patterns among DCN subnuclei. The differential regulation of IEGs such as *Fos*, *Junb*, *Egr1*, and *Npas4* underscores their unique contributions to fear learning and memory processes within the cerebellum. These findings highlight region-specific neuronal activation in the DCN and suggest that IEGs orchestrate distinct molecular pathways critical for the formation and retrieval of fear memories.

### snRNA-seq in the DCN reveals cell type- and learning phase-specific transcriptional changes

**Figure 5:**
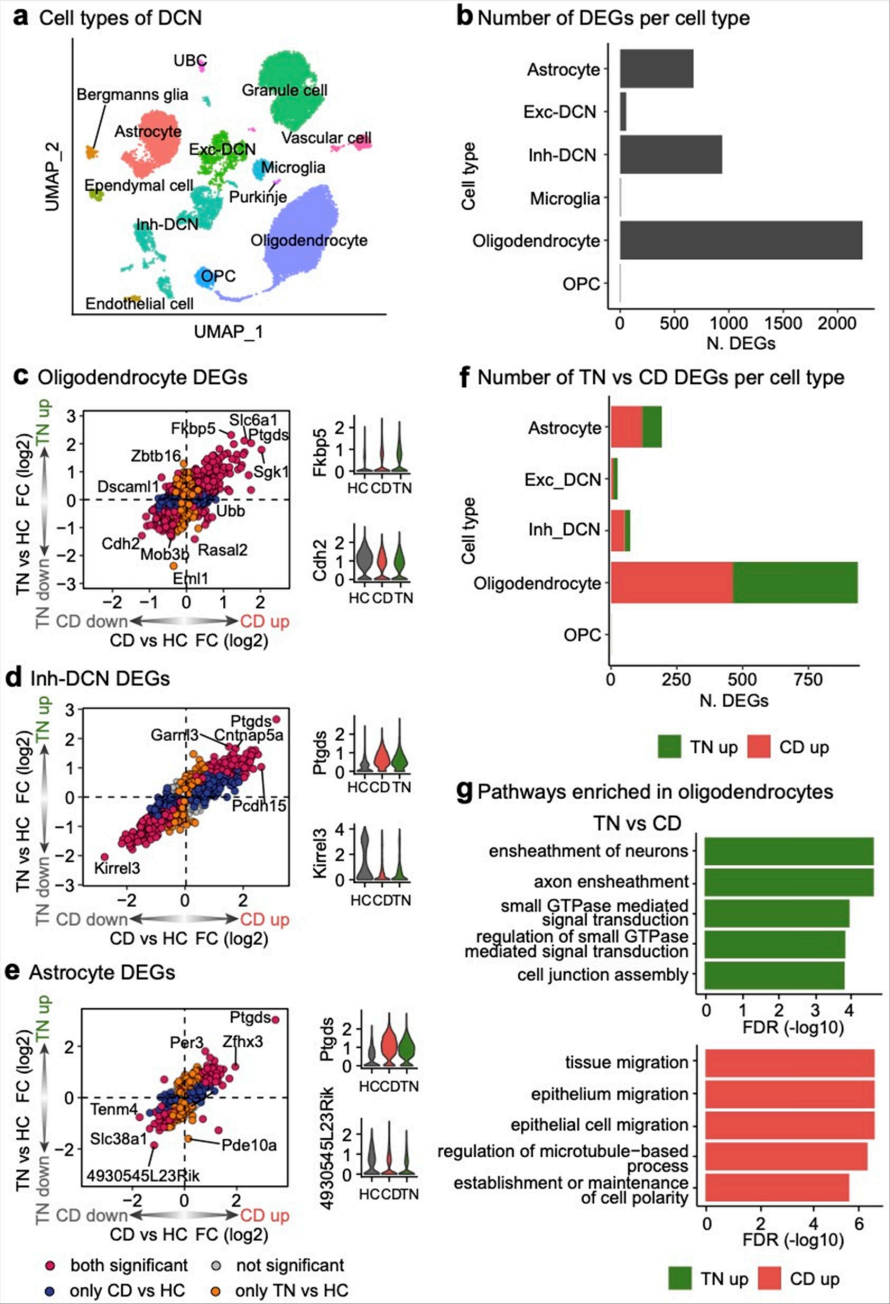
Cell type-specific transcriptional changes within the DCN during fear learning **a**, UMAP representation of the snRNA-seq dataset, colored by cell types (24,290 nuclei). Exc-DCN, excitatory neurons in DCN; Inh-DCN, inhibitory neurons in DCN. **b**, Bar plot showing the number of significant LRT DEGs per cell type (adjusted *P* < 0.05). **c–e**, Quadrant plots showing log_2_FC values for oligodendrocyte (**c**), Inh-DCN (**d**), and astrocyte (e) DEGs in the CD vs. HC (x-axis) and TN vs. HC comparisons (y-axis). Genes are colored based on the comparison in which they are significant. Violin plots display the normalized expression patterns of a “both significant” DEG with the highest log_2_FC in each quadrant I (left) and quadrant III (right), separated by condition. **f**, Bar plot showing the number of significant TN vs. CD DEGs per cell type (adjusted *P* < 0.05). **g**, GO enrichment analysis results for TN vs. CD DEGs in oligodendrocytes, categorized by biological processes. Bar colors indicate the direction of enrichment, and FDR values represent significance.

The DCN consists of various cell types (Fig. 1c) that contribute to complex neurological processes such as fear learning. To better understand the role of individual cell types in the learning and retrieval of fear memories, we performed snRNA-seq on DCN samples collected from mice under the same experimental conditions as before (HC, CD, and TN). Considering the rapid translocation of mRNA to the cytoplasm, the DCN was dissected 30 min after either fear conditioning or tone retrieval. After excluding cell types predicted to be contaminants from adjacent regions, six distinct cell types were identified based on the expression of canonical marker genes (Fig. 5a, **Extended Data** Fig. 4a).

Among these, oligodendrocytes exhibited the highest number of significant LRT DEGs (n = 2,106), followed by inhibitory neurons (n = 944) and astrocytes (n = 728). (Fig. 5b). Pairwise comparisons for each cell type revealed distinct DEG patterns, highlighting that fear learning involves interactions between various cell types with specialized roles (Fig. 5c–e, **Supplementary Table 4a**).

Interestingly, DEG analysis comparing the TN vs. CD conditions revealed that oligodendrocytes exhibited a significantly higher number of DEGs than other cell types, such as inhibitory neurons and astrocytes, which showed relatively few DEGs (Fig. 5f, **Supplementary Table 4a**). This pronounced transcriptional activity in oligodendrocytes was much less evident in the CD vs. HC and TN vs. HC comparisons (**Extended Data** Fig. 4b), suggesting that oligodendrocytes are primarily involved during fear memory storage. This observation contrasts with previous assumptions that memory retrieval involves substantial glial-neuron interactions^45^. GO enrichment analysis of the upregulated DEGs in the TN phase compared to the CD phase showed strong associations with myelination-related processes (Fig. 5g, **Supplementary Table 4b**), consistent with recent studies demonstrating that new myelin formation supports the preservation of remote fear memory recall^46,47^. To identify the transcriptional regulators driving these gene expression changes, we conducted a transcription factor (TF) analysis. The most highly activated TF in oligodendrocytes during the TN phase compared to the CD phase was *Smad3* (**Extended Data** Fig. 4c, **Supplementary Table 4c**). *Smad3*, known to regulate the timing of central nervous system myelination via TFG-beta signaling^48^, emerged as a key regulator of fear memory retrieval in oligodendrocytes.

#### Specific inhibitory neuron subtype expressing Kit is highly associated with fear conditioning

**Figure 6:**
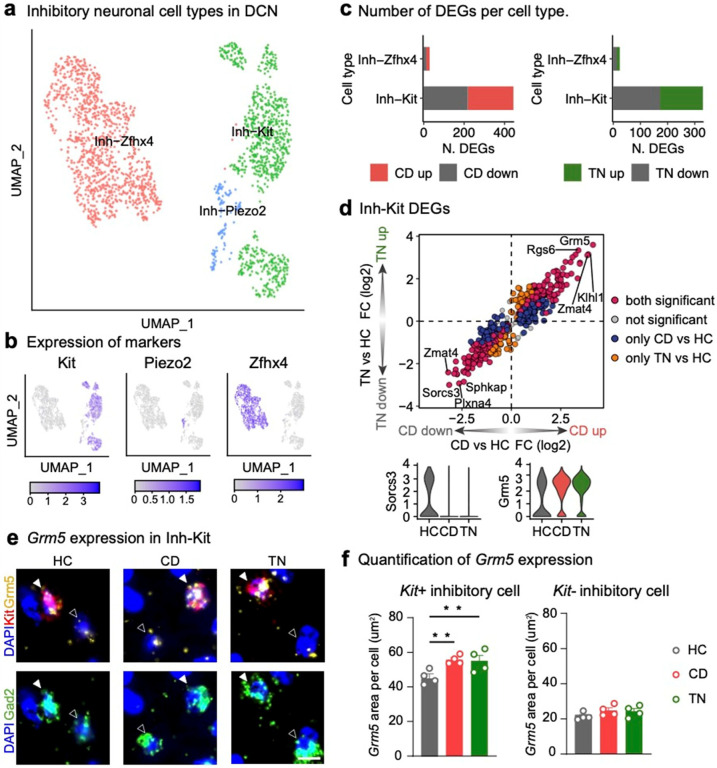
Inh-Kit neurons show significant changes in response to fear conditioning **a**, UMAP representation of inhibitory neurons in the DCN, colored by subtype (1,980 nuclei). **b**, UMAP representation of subtype marker gene expression (*Kit*, *Piezo2*, and *Zfhx4*). **c**, Bar plots showing the number of significant DEGs per inhibitory neuron subtype for the CD vs. HC (right) and TN vs. HC comparisons (right). Significant DEGs were defined as adjusted *P* < 0.05 and log_2_FC > ±0.1. **d**, Quadrant plot showing log_2_FC values for Inh-Kit DEGs in the CD vs. HC (x-axis) and TN vs. HC comparisons (y-axis). Genes are colored based on the comparison in which they are significant. Violin plots display the normalized expression patterns of a “both significant” DEG with the highest log_2_FC in each quadrant I (left) and quadrant III (right), separated by condition. **e**, ISH results showing the expression of *Grm5* in the DCN after fear learning. Filled arrows indicate *Gad2*+ *Kit*+ cells (*Kit*+ inhibitory cells) and open arrows indicate *Gad2*+ *Kit*− cells (*Kit*− inhibitory cells). Scale bar: 10 μm. **f**, Quantification of ISH results (n = 4 per group). *Kit*+ inhibitory cells: One-way ANOVA: F(2,9) = 6.007, *P* = 0.0220. Dunnett’s multiple comparisons test: *P = 0.0233 (HC vs. CD), *P = 0.0314 (HC vs. TN). *Kit*− inhibitory cells: One-way ANOVA: F(2,9) = 1.526, *P* = 0.2687. Dunnett’s multiple comparisons test: *P* = 0.2320 (HC vs. CD), *P = 0.3323 (HC vs. TN).

In our analysis, inhibitory neurons (Inh-DCN) exhibited more DEGs than excitatory neurons (Exc-DCN) (Fig. 5b). Given the significant heterogeneity within inhibitory neurons^49^, we further analyzed these cells by categorizing their subtypes. Based on the classification by Kebschull *et al*., inhibitory neurons in the DCN can be grouped into three subtypes characterized by specific marker genes: *Zfhx4*, *Kit*, and *Piezo2*^10^. To explore this heterogeneity, we performed unsupervised clustering of inhibitory neurons in our snRNA-seq dataset, identifying eight clusters that predominantly expressed one of these three marker genes (**Extended Data** Fig. 5a, b). Using this expression pattern, we classified the clusters into three subtypes: Inh-Zfhx4, Inh-Kit, and Inh-Piezo2 (Fig. 6a, b).

We performed subtype-specific DEG analyses across three conditions (CD vs. HC, TN vs. HC, and TN vs. CD). Among the subtypes, Inh-Kit displayed a significantly higher number of DEGs in both the CD vs. HC and TN vs. HC comparisons (Fig. 6c, **Supplementary Table 5a**). Despite the similar proportions of the Inh-Kit and Inh-Zfhx4 subtypes among inhibitory neurons in the DCN (**Extended Data** Fig. 5c), the pronounced transcriptional activity in Inh-Kit suggests a critical role in the fear learning process. Interestingly, the ratio of *Kit* + inhibitory neurons (*Kit* + *Gad2* + cells) among total inhibitory neurons (*Gad2*+) varied across DCN subnuclei. However, fear conditioning did not affect the overall population of inhibitory neurons or *Kit* + inhibitory neurons (**Extended Data** Fig. 6a–d). This indicates that the three subnuclei have distinct cell type compositions, suggesting specialized roles for each subnucleus in fear memory processing. Among the top DEGs, *Grm5* was the most upregulated gene in Inh-Kit during both the CD and TN phases compared to the HC phase (Fig. 6d). *Grm5* encodes the metabotropic glutamate receptor mGluR5, which is crucial for neuronal plasticity^50,51^. Fear acquisition and extinction are impaired in mGluR5 knockout mice^52^, underscoring its role as a key regulator of fear learning and memory^53,54^.

To validate the subtype-specific expression of *Grm5*, we performed ISH and confirmed that *Grm5* was significantly upregulated in *Kit* + inhibitory cells, but not in *Kit*− inhibitory cells, following fear conditioning (Fig. 6e, f, **Extended Data** Fig. 6e). Notably, this upregulation persisted during the retrieval phase, indicating the sustained involvement of *Grm5* in fear learning and memory processes.

TF analysis revealed highly cell type-specific TF activity changes in Inh-Kit (**Extended Data** Fig. 5d, **Supplementary Table 5b**). Among the top TFs activated during both the CD and TN phases, Thrb was identified as a key regulator of *Grm5*. Thyroid hormones, encoded by *Thrb*, are essential for normal brain development, and their deficiency has been linked to intellectual and neurological impairments^55^. Our results highlight the unique role of Inh-Kit inhibitory neurons in fear learning and suggest key transcriptional changes underlying their function.

## Discussion

In this study, we demonstrate that classical fear conditioning induces region-, cell type-, and learning phase-specific transcriptomic changes in the cerebellum. Spatial transcriptomic analysis revealed distinct transcriptional profiles across different cerebellar regions during various stages of fear learning. Our findings underscore that the role of the cerebellum in fear learning is not uniform but instead involves specialized and coordinated transcriptional changes in specific circuits. Notably, the robust induction of IEGs (*Fos*, *Junb*, and *Egr1*) in the DCN was observed, highlighting the significance of this output hub in shaping fear memory processes. Interestingly, vermis displayed broader transcriptional changes than the hemisphere, consistent with its established role in integrating conditioned and unconditioned stimuli. In the DCN, where region-specific activation appeared, oligodendrocytes and specific inhibitory neuron subtypes, such as *Kit* + neurons, exhibited unique transcriptional responses, suggesting that glial and inhibitory circuits contribute distinctively to encoding and retrieving fear memories. These findings expand our understanding of cerebellar functions beyond motor coordination and suggest potential molecular targets for exploring cellular mechanisms underlying fear learning.

IEGs such as *c-Fos*, *Junb*, *Egr1*, and *Npas4* are well-established memory tags in brain regions such as the hippocampus, medial frontal cortex, and amygdala^56–58^; however, their roles in cerebellar learning mechanisms remain underexplored^4,32^. Here, we observed robust fear-conditioning-dependent upregulation of IEGs in the DCN, suggesting their role as memory tags in cerebellar circuits^59^. Each IEG exhibited distinct expression patterns across fear learning stages and DCN subnuclei. Given that IEGs also function as transcription factors targeting specific genes^60^, our findings suggest they may play specialized roles in fear learning^61–64^. However, IEG expression was not detected in our snRNA-seq dataset, likely due to technical limitations; the Visium platform captures transcripts in tissue spots, whereas snRNA-seq focuses on nuclear RNA. By the time of brain dissection, IEG mRNAs may have been exported to the cytoplasm, leaving their cell type of origin unresolved.

The cerebellar vermis exhibited more extensive transcriptional changes than the hemisphere across all stages of fear learning. This finding aligns with that of previous studies identifying the vermis as a core site for the convergence of conditioned and unconditioned stimuli during classical fear conditioning, regulating fear-evoked freezing behavior and bradycardia^6,23–25,65,66^. Our findings extend this understanding by showing that the vermis undergoes distinct transcriptomic remodeling throughout multiple learning phases.

We identified *Ttr* and *Selenow*, genes implicated in oxidative stress and energy metabolism^67–70^, as highly upregulated in the vermis following tone memory retrieval. These genes may regulate the metabolic states required for learning-induced changes. Interestingly, their upregulation appeared primarily during the memory consolidation phase, as their expression was significantly higher during tone retrieval than during training. However, our analysis focused primarily on coronal sections encompassing lobules V and VI, leaving the possibility of transcriptional changes in other cerebellar regions, such as lobule VIII^71,72^, unexplored.

Oligodendrocytes in the DCN displayed the highest number of DEGs across fear conditioning phases, with pronounced transcriptional changes between memory consolidation and retrieval, a period during which other cell types showed minimal or no transcriptional changes. During the TN phase, the expression of genes related to myelination was significantly higher than that in the CD phase. Oligodendrocyte-mediated myelination supports rapid and stable neuronal communication, which is crucial for synaptic plasticity^73^. These findings align with those of a previous study showing that the DCN–lPBN circuit undergoes synaptic potentiation during auditory fear conditioning^22^.

A classical understanding of the interaction between the cerebellar cortex and DCN is that Purkinje cells in the cerebellar cortex alter their firing rates and synchronization, exerting inhibitory inputs to the DCN to modulate the firing rates of DCN neurons, which in turn regulate DCN output signals to frontal brain regions^74,75^. DCN neurons exhibit diverse projection patterns, and emerging evidence suggests the presence of multiple distinct cell types within the DCN^10,76–81^. Here, we identified *Kit* + inhibitory neurons in the DCN as undergoing prominent transcriptional changes during classical fear conditioning. Notably, the *Grm5* gene, encoding mGluR5, was significantly upregulated in *Kit* + inhibitory neurons during both fear acquisition and retrieval phases. This suggests that, besides modulating DCN firing, these neurons engage in learning-dependent synaptic plasticity, potentially forming an inhibitory engram. Our work provides new insights into the role of the cerebellum in non-motor associative learning, highlighting region-, cell type-, and learning phase-specific transcriptional changes. The identification of IEGs in the DCN raises the exciting possibility of experimentally probing cerebellar fear memory engrams. Future research should focus on dissecting the glial and inhibitory circuits involved in cerebellar learning to further elucidate the contribution of the cerebellum to learning and memory.

## Online Methods

### Animals

6 to 8-week aged male wild-type mice (C57Bl/6N from Orient Bio Inc., Seongnam, Korea), were purchased and acclimated to the facility for at least one week before the experiment. All mice were maintained on a 12 h light/dark cycle with *ad libitum* access to food and water. All experiments were performed in accordance with the guidelines approved by the Institutional Animal Care and Use Committee of Seoul National University (SNU-221014–3).

### Fear conditioning

For fear conditioning, mice were placed in fear conditioning chamber. After 2min exploration period, mice received five CS (30 s, 2800 Hz, 79 dB tone) and US (2 s, 0.5 mA, electrical foot shock) pairings. For tone retrieval test, mice were placed in a novel chamber (cylinder chamber) 24h after conditioning. 2 or 3 CS with a 30-s interval were represented. All behavior tests were performed between 09:00 and 16:00 during the light phase of the light/dark cycle.

### Sample preparation for spatial transcriptomics

60 min after fear conditioning or retrieval test, mice were anesthetized with isoflurane and perfused with normal saline. Extracted brains were flash frozen in isopropyl alcohol at −70°C. Frozen brains were embedded in optimal cutting temperature (OCT) compound (VWR, Cat No. 25608–930) and were stored at −80°C until sectioning. Brains with a RIN (RNA integrity and Number) above 7 were used.

Tissue blocks were equilibrated to − 15°C and sectioned at a thickness of 10 μm using cryostat (Thermo, Cryostar NX70). The sections were mounted onto the active capture areas of 10x Genomics Visium slides (10x Genomics, PN 1000184) and stored in airtight containers at − 80°C until Visium library preparation. Prior to Visium library preparation, methanol fixation and H&E (hematoxylin and eosin) staining were performed following the Visium protocol (10x Genomics, CG000160).

Visium libraries were constructed following the manufacturer’s instructions (10x Genomics, CG000239). Sequencing was performed on a Novaseq 6000 platform (Illumina, Cat No. 20012850) using S1 sequencing reagents (Illumina, Cat No. 20028318), targeting 120 million reads per library using dual indexing. The resulting FASTQ files were aligned to the mm10 reference genome using Space Ranger (10x Genomics, v1.3.1).

### In situ hybridization (RNAscope)

After fear conditioning or retrieval test, mice were anesthetized with isoflurane 60 min after for spatial transcriptomics validation and 30 min after for snRNA-seq. Whole brains were obtained and flash-frozen in isopentane equilibrated on dry ice. Frozen brains were embedded in OCT and maintained at −80°C until sectioning. 14-μm coronal sections including DCN were collected on Superfrost slides and stored at −80°C until processing. Multi-channel fluorescence in situ hybridization was performed using the RNAscope^™^ Multiplex Fluorescent Reagent Kit v2 (Advanced Cell Diagnostics) following manufacturer’s instructions. In brief, slides were fixed in 4% PFA and serial dehydration step was done in ethanol. Then, hydrogen peroxide treatment, followed by protease 4 treatment was done at room temperature. Hybridization was done at 40°C for 2h. Following probes were used: *Fos* (316921-C2), *Junb* (556651-C1), *Egr1* (423371-C2), *Npas4* (423431-C3), *Eef1a2* (1262661-C1), *Kit* (314151), *Grm5* (423631-C2), Gad2 (439371-C3). The slides were imaged using slide scanner (Zeiss, Axio Scan Z1). We used ABBA workflow provided by https://biop.github.io/ijp-imagetoatlas/ for ROI registration, then analyzed with ImageJ and Qupath^82^.

### DCN sample preparation for snRNA-seq

30min after fear conditioning or retrieval test, mice were anesthetized and decapitated immediately. Brains were extracted and sectioned using a vibratome (VT1200S, Leica) in pre-chilled ACSF. The DCN were quickly micro-dissected from 300 μm of cerebellar coronal slices and stored at −80°C.

Visium spatial transcriptomics analysis

### Spatial transcriptomics mapping and preprocess

Space Ranger (v1.3.1) analysis pipelines were used to align raw FASTQ files to mm10 reference genome and image files.

Output files were loaded onto Scanpy v1.10.0^83^ as anndata objects for further preprocessing. We removed doublets using Scrublet v0.2.3^84^. *Gm42418* and genes expressed in less than 3 nuclei were removed. Nuclei that have fewer than 100 genes, more than 8,000 genes, and more than 50,000 UMI counts were filtered out. Spots with over 35% mitochondrial gene expression were also excluded. Raw gene read counts of each sample were normalized for library size correction and then log-transformed.

To account for spot similarity in both feature and spatial space, the clustering process was conducted in two main stages. First, single-cell variational inference (scVI) v1.1.2^85^ was used to adjust batch effects between each sample. The top 2,000 most highly variable genes of the merged dataset were identified using sc.pp.highly_variable_genes() with “Seurat v3” flavor and used for scVI analysis. The resulting latent representation was used for neighbor identification, which estimates ‘connectivities’ of spots based on gene expression. Next, the spatial connectivity matrix of spots was calculated using Squidpy v.1.2.2^86^. The two connectivity graphs were added to form a joint graph, with a 0.8 importance weight for the gene expression connectivity and 0.2 importance weight for the spatial connectivity. Clustering of spots was performed on the joint connectivity graph using the Leiden algorithm with a resolution of 0.8.

### Annotation of spatial clusters

We used cell2location v.0.1.3^87^ to map cerebellum cell types on our Visium data. To account for only cerebellum regions, we subset spatial clusters 1,2,3,4,5, and 9 that are within the cerebellum, as guided by anatomical structures. Cerebellum scRNA-seq data from the Allen Brain Cell Atlas^28^ was utilized as the reference data. For the reference data, only genes detected in more than 5 cells, more than 3 percent of cells, and average expression in non-zero cells higher than 1.12 were retained.

Cell2location estimates reference gene signatures of cell types from reference scRNA-seq using negative binomial regression and accounting for batch effects. Reference model was built using ‘donor_label’ as the batch key. By mapping our query Visium dataset on the model, the absolute spatial abundance of each reference cell type was estimated for each spot. For parameters, the expected cell abundance for each spot (N_cells_per_location) was set to 6, the detection alpha was set to 20, and the number of training epochs was set to 30,000.

To annotate each spatial cluster using cell2location cell type abundance results, the following approach was utilized: Cell abundance values for each cell type were binarized using the 99th quantile, and the binarized labels were assigned to each spot. Fisher’s exact test was conducted using the binary labels for each spatial cluster. Each cluster was annotated with the cell type exhibiting the highest odd ratio (OR) among the region-specific cell types, which included DCN, molecular layer, granular layer, and Purkinje cell layer.

Spatial clusters that could not be annotated to a particular region using the above analysis were further classified using anatomical knowledge. Cluster 4, located below the cerebellar cortex (**Extended Data** Fig. 1a) and showing high enrichment for glial cell types (Fig. 1c), was identified as white matter. Cluster 9, which exhibited the highest enrichment for inferior colliculus (IC) (Fig. 1c) - a region not present in our obtained image slices - was annotated as molecular layer through manual comparison with Allen Brain Atlas *in situ* hybridization data^29^. Clusters 0,6,7,8,10,11, and 12, located in the medulla region, were also annotated through manual comparison with *in situ* hybridization reference data.

We manually annotated the Purkinje layer into vermis and hemisphere subregions using an anatomical atlas based on the Allen Brain Atlas. Spots located in lobules 4/5, lobule 6, and the simplex lobule were classified as part of the vermis region, while spots in Crus I, Crus II, and the surrounding regions were categorized as the hemisphere region.

### Differential gene expression analysis

Both single-cell based and pseudo-bulk method was conducted for DEG analysis. Results from the single-cell based method suggested gene expression variability within the sample. To mitigate this variability, we determined that the pseudo-bulk approach will be more appropriate for our dataset.

For Purkinje cell layer subregions, we used DeSeq2 v.1.40.1.^88^ on pseudo-bulk samples for DEG analysis. For each subregion and sample, we created 2 pseudo-replicate samples by dividing the spots within that group into 2 subsets of roughly equal sizes, then summing up transcript counts for each gene. Spatial cluster – sample pairs with less than 10 spots were excluded. Genes with expression in less than 3 pseudo-replicates and mitochondrial genes were removed for the analysis.

Initially, we tested the significance of genes at any level of the condition factor using “LRT” as the test parameter in the DESeq() function. Next, we determined the DEGs for each pairwise comparison - CD vs HC, TN vs HC, and TN vs CD - using the standard DESeq() function with parameters alpha as 0.05 and independentFiltering set to false. In consideration of the small number of spots in each subregion, we repeated this process 100 times, with different seeds for every pseudo-replicate creation. Genes that were significant (adjusted *P* < 0.05) in more than 80% of the LRT tests were considered significant LRT DEGs. Significant LRT DEGs with adjusted *P* < 0.05 and log_2_FC > ± 0.1 in more than 80% of the pairwise comparison tests were considered significant DEGs.

DEGs from the comparison between the vermis and hemisphere subregions were identified using the same method, excluding the LRT analysis.

The DEG analysis for each cerebellum region followed the same method as the Purkinje subregion but with adjusted criteria: 3 pseudo-replicates were created, a minimum of 10 spots was required for spatial cluster–sample pairs, and the gene expression threshold was set at 5 pseudo-replicates. The analysis process was conducted only once for each region. Genes that were significant in the LRT, with adjusted *P* < 0.05 and log_2_FC > ± 0.1, were considered significant DEGs.

### Pathway enrichment analysis

GO biological processes enriched for region-specific DEGs were analyzed using clusterProfilter v4.10.0^89^. Significantly enriched terms were identified using a threshold of Benjamini-Hochberg corrected *P* < 0.05.

snRNA-seq data analysis

### Preprocess and annotation

We used cellranger (v7.1.10) analysis pipelines to align raw FASTQ files to mm10 reference genome.

To proceed with further preprocessing, the output files were imported into Scanpy v1.10.0^83^ as an anndata object. Doublets were called and excluded using Scrublet^84^. Count matrix was filtered for genes expressed in less than 3 nuclei and the *Gm42418* gene which is associated with ribosomal RNA contamination. Nuclei with fewer than 200 genes, more than 8,000 genes, more than 60,000 UMI counts, and mitochondrial contents over 20% were excluded. Normalization and log-transformation of the raw gene read counts were applied to stabilize variance across expression values.

scVI v1.1.2^85^ was used to adjust batch effects between each sample, with top 2,000 most highly variable genes of the merged dataset identified using sc.pp.highly_variable_genes() with “Seurat v3” flavor and used for scVI analysis. The resulting latent representation was used for neighbor identification, and clustering of nuclei was performed using the Leiden algorithm with a resolution of 1. Annotation of each cluster was conducted by comparing marker genes with well-known cell-type marker genes from various references^15,28,29,90^.

### Differential gene expression analysis

To identify DEGs between HC, CD, and TN conditions per cell type, we used MAST v1.28.0^91^. Only genes expressed in more than 5% of the nuclei per cell type were included in the analysis. We used the zlm function to fit our gene expression data to a Hurdle model with the cellular detection rate (proportion of genes that have expression within a single nuclei) as a covariate. Initially, we ran an LRT using MAST’s latest function to test which genes are differentially expressed when we drop the ‘condition’ factor. Genes with adjusted *P* < 0.05 were considered significant LRT DEGs. Next, we conducted DEG analysis for each pairwise comparison of fear learning phases: CD vs. HC, TN vs. HC, TN vs. CD. Genes that are significant LRT DEGs and pass the adjusted *P* < 0.05 and log_2_FC > ± 0.1 threshold were considered significant DEGs for each pairwise comparison. GO biological processes enriched for cell type-specific DEGs were identified using clusterProfilter v4.10.0^89^.

### Transcription factor activity analysis

Python-based single-cell regulatory network inference and clustering (pySCENIC) package^92^ v0.12.1 was used to estimate TF activity and its target genes for each nuclei. Briefly, adjacency between a TF and its targets was calculated using the GRNBoost2 algorithm^93^. Next, to identify TF-enriched motifs and their target genes, two gene motif ranking datasets (mm10_10kbp_up_10kbp_down_full_txt_v10 and mm10_500bp_up_100bp_down_full_tx_v10) from the RcisTarget database were used. Finally, AUC enrichment scores were predicted for each nuclei to quantify the activity of TFs.

We evaluated the significant change of TF activity between two conditions for each cell type with a one-sided Wilcoxon rank-sum test on regulon activity scores (RAS), using rstatix v0.7.2. To exclude TFs with low activity, only TFs with an average RAS within the cell type higher than the dataset-wide average were considered. Bonferroni correction was applied, and TFs with adjusted *P* < 0.01 were considered significantly differentially regulated.

## Figures and Tables

**Figure 4:**
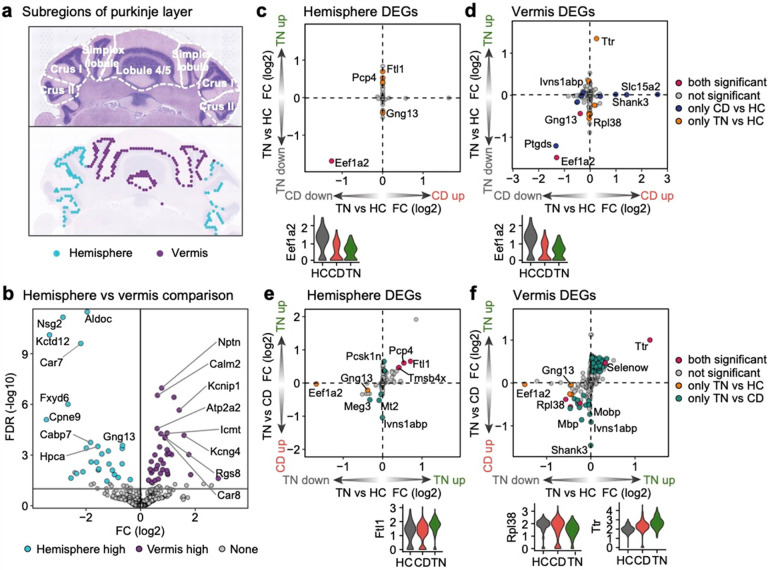
Subregion-specific analysis of the Purkinje cell layer across fear learning stages **a**, Anatomical (top) and spatial (bottom) plots of the Purkinje cell layer. The anatomical plot is divided based on cerebellar lobules, referencing the Allen Brain Atlas. Purkinje cell layer spots in the spatial plot were manually split according to the anatomical plot. The hemisphere includes Crus I and Crus II, while the vermis includes the simplex lobule, lobules 4/5, and lobule 6 (lobule 6 is included in the HC sample; see **Extended Data Fig. 2b**). **b**, Volcano plot showing differentially expressed genes (DEGs) in a hemisphere vs vermis comparison. Significant DEGs were defined as adjusted *P* < 0.05 and log_2_FC > ±0.1. All three condition samples were used for the analysis. **c–f**, Quadrant plots illustrating log_2_(fold change) (log_2_FC) values for the CD vs. HC (x-axis) and TN vs. HC (y-axis) comparisons in the hemisphere (**c**) and vermis (**d**) regions, and for the TN vs. HC (x-axis) and TN vs. CD (y-axis) comparisons in the hemisphere (**e**) and vermis (**f**) regions. Genes are colored based on the comparison in which they are significant. Violin plots display the normalized expression patterns of a “both significant” DEG with the highest log_2_FC in each quadrant I (left) and quadrant III (right), separated by condition.
